# Supplementary material for: Human milk-based fortifier is associated with less alteration of milk fat globule size than cow milk-based fortifier
Source: PLoS One. 2021 Dec 7;16(12):e0257491. doi: 10.1371/journal.pone.0257491 (PMC8651125; doi:10.1371/journal.pone.0257491)
Supplement: Attachment — Submitted filename: Responses to reviewers, human milk-based fortifier study.docx [file pone.0257491.s003.docx]

Journal Requirements:

When submitting your revision, we need you to address these additional requirements.

1. Please ensure that your manuscript meets PLOS ONE's style requirements, including those for file naming. The PLOS ONE style templates can be found at <https://journals.plos.org/plosone/s/file?id=wjVg/PLOSOne_formatting_sample_main_body.pdf> and <https://journals.plos.org/plosone/s/file?id=ba62/PLOSOne_formatting_sample_title_authors_affiliations.pdf>

Updated to match requirements

2. To comply with PLOS ONE submission guidelines, in your Methods section, please provide additional information regarding your statistical analyses. For more information on PLOS ONE's expectations for statistical reporting, please see <https://journals.plos.org/plosone/s/submission-guidelines.#loc-statistical-reporting>.

Added to the manuscript.

3. Please provide additional details regarding participant consent. In the ethics statement in the Methods and online submission information, please ensure that you have specified (1) whether consent was informed and (2) what type you obtained (for instance, written or verbal, and if verbal, how it was documented and witnessed). If your study included minors, state whether you obtained consent from parents or guardians. If the need for consent was waived by the ethics committee, please include this information.

We obtained written, informed consent from all mothers to use their milk for research purposes (added to the manuscript). We did not include any minors.

If you are reporting a retrospective study of medical records or archived samples, please ensure that you have discussed whether all data were fully anonymized before you accessed them and/or whether the IRB or ethics committee waived the requirement for informed consent. If patients provided informed written consent to have data from their medical records used in research, please include this information.

We did not include a retrospective chart review or utilized archived samples as part of this study.

4. Thank you for stating the following in the Financial Disclosure section:

'KH receives speaker honoraria from Prolacta Bioscience.
Prolacta Bioscience ([www.prolacta.com](http://www.prolacta.com/)) provided the product for use in this study but was not involved in the study design, data collection or analysis, performance of the study, outcomes, decision to publish, or manuscript writing.'

We note that you received funding from a commercial source: Prolacta Bioscience

Please provide an amended Competing Interests Statement that explicitly states this commercial funder, along with any other relevant declarations relating to employment, consultancy, patents, products in development, marketed products, etc.

Within this Competing Interests Statement, please confirm that this does not alter your adherence to all PLOS ONE policies on sharing data and materials by including the following statement: "This does not alter our adherence to PLOS ONE policies on sharing data and materials.” (as detailed online in our guide for authors http://journals.plos.org/plosone/s/competing-interests).  If there are restrictions on sharing of data and/or materials, please state these. Please note that we cannot proceed with consideration of your article until this information has been declared

Please include your amended Competing Interests Statement within your cover letter. We will change the online submission form on your behalf.

We have included the appropriate Competing Interests Statement and information within our updated cover letter.

5. Please upload a copy of Figures 1 to 4, to which you refer in your text. If the figures are no longer to be included as part of the submission please remove all reference to them within the text.

Thank you, Figures 1-4 will be uploaded.

. Review Comments to the Author

Please use the space provided to explain your answers to the questions above. You may also include additional comments for the author, including concerns about dual publication, research ethics, or publication ethics. (Please upload your review as an attachment if it exceeds 20,000 characters)

Reviewer #1: This study aimed to assess the change of the MFG size with the fortification of a human milk-based or a cow milk-based fortifier. Changes in the particle size might associate with feeding intolerance. This is of great relevance, particularly for low-birth-weight or preterm infants.

Over the manuscript, there are some inconsistencies in the grammar, as well as some repetitiveness in some parts. I would suggest authors revise the manuscript.

A native English speaker has provided writing and editing assistance for the manuscript to eliminate errors and repetitiveness.

The Introduction section seems very broad. In this part, the authors should describe more about the relevance of the MFG size, the differences in composition of the two fortifiers, etc. This would create a greater interest in the reader.

More information about the MFG size has been added to the introduction. A comparison of the 2 fortifiers has been added in Table 1.

Regarding the methodology, was the MGF size measurement done in duplicate, or were they single measurements?

The MFG size was measured three times for each sample.

What software was used to conduct the statistical analyses?

All statistical analyses were performed using NCSS 2021 Statistical Software (2021, NCSS, LLC. Kaysville, Utah, USA).

Could the author summarize the demographic characteristics of the donors (e.g. age, weight, BMI)?

Samples of human milk were obtained from 9 Japanese women who were 27-36 years of age and 3-6 months postpartum. This has been added to the Results section.

We did not obtain other demographic information such as weight or BMI.

The results section mentions three different figures which were not part of the manuscript. I wonder if there was an issue with the website/system. I can not discuss much about the information associated with the figures.

We are sorry for not having attached the figures. In revised manuscript, we confirmed that we could upload the figures appropriately.

Table 1 could include more information to make it more comprehensive. For example, could you add the number of samples in each group (n=), as well as the p values for the differences within and between groups?

We added the number of samples in each group as well as the p values for the differences in Table 2.

The discussion summarizes the findings and the impact on MFG sizes. Although the authors discuss some of the possible mechanisms, they could further explain how human milk-based or a cow milk-based fortifier changes the size and structure of the MFG and how the consumption affects the feeding tolerance in infants consuming these fortifiers.

The alteration of the microstructure of MFG with cold temperatures and with a cow milk-based fortifier in part relates to poorer digestion ability. We are uncertain of the mechanism causing this effect. More data is needed to determine this mechanism of action.

Reviewer #2: Review Comments to the Author
This paper aimed at to evaluate the HMBF and CMBF on the MFG, and to explain it impact on feeding tolerance. The result sounds interesting, however, the paper still explain the detail of the test and how it works, more data need before it could accept. Question is list below:
1. For the particle size of fat globule, many parameter many effect the result, such as pH, Ca and casein content in milk. You should add this data in the paper.

Thank you for this helpful comment. We have added the data on pH, calcium, osmolarity and casein content in fortified milk for both types of fortifiers to Table 1.

2. Line 76-80, you collected human milk from 9 mother, do you mixed the 9 sample together or Separated? If you separate, does all sample the protein is 2.9g/100ml? for the line 79, more information about CMBF is need, company, composition.

We did not mix the samples. We fortified the donor milk according to the manufactures’ recommendation. The protein content 2.9g/100ml was estimated from the average protein content of human milk; we deleted this part in the revision.

3. Line 82-83, after you mixed together, you test the size in 0, 1, 24, 48h. Does the sample is put in refrigerator? What’s temperature you keep the sample?

Yes, we kept the sample in a refrigerator at 4℃

4. Excepted the particle size, such as the scanning electron microscope is need to show the difference of the samples.

We performed the electron microscope experiment as the reviewer suggested and it shows the difference in MFG size visually. We appreciate this suggestion.

5. The figures is not show in the paper, please added it.

We apologize for not having attached the figures. Now attached.

6. PLOS authors have the option to publish the peer review history of their article ([what does this mean?](https://journals.plos.org/plosone/s/editorial-and-peer-review-process#loc-peer-review-history)). If published, this will include your full peer review and any attached files.
